# Do nuclear-encoded core subunits of mitochondrial complex I confer genetic susceptibility to schizophrenia in Han Chinese populations?

**DOI:** 10.1038/srep11076

**Published:** 2015-06-08

**Authors:** Xiao Li, Wen Zhang, Jinsong Tang, Liwen Tan, Xiong-jian Luo, Xiaogang Chen, Yong-Gang Yao

**Affiliations:** 1 Key Laboratory of Animal Models and Human Disease Mechanisms of the Chinese Academy of Sciences & Yunnan Province, Kunming Institute of Zoology, Chinese Academy of Sciences, Kunming 650223, China; 2 Institute of Mental Health, the Second Xiangya Hospital, Central South University, Changsha 410011, China; 3 Kunming College of Life Science, University of Chinese Academy of Sciences, Kunming 650223, China; 4 CAS Center for Excellence in Brain Science, Chinese Academy of Sciences, Shanghai, 200031, China

## Abstract

Schizophrenia is one of the most prevalent psychiatric disorders with complex genetic etiology. Accumulating evidence suggests that energy metabolism and oxidative stress play important roles in the pathophysiology of schizophrenia. Dysfunction of mitochondrial respiratory chain and altered expression of complex I subunits were frequently reported in schizophrenia. To investigate whether nuclear-encoded core subunit genes of mitochondrial complex I are associated with schizophrenia, we performed a genetic association study in Han Chinese. In total, 46 tag single nucleotide polymorphisms (SNPs) from 7 nuclear-encoded core genes of mitochondrial complex I were genotyped in 918 schizophrenia patients and 1042 healthy controls. We also analyzed these SNPs in a large sample mainly composed of Europeans through using the available GWAS datasets from the Psychiatric Genomics Consortium (PGC). No significant associations were detected between these SNPs and schizophrenia in Han Chinese and the PGC data set. However, we observed nominal significant associations of 2 SNPs in the *NDUFS1* gene and 4 SNPs in the *NDUFS2* gene with early onset schizophrenia (EOS), but none of these associations survived the Bonferroni correction. Taken together, our results suggested that common SNPs in the nuclear-encoded core subunit genes of mitochondrial complex I may not confer genetic susceptibility to schizophrenia.

Schizophrenia is one of the most common psychiatric disorders with a heritability as high as 80%^1^. Though significant progress has been made during the past decades, the etiology and pathophysiology of schizophrenia remains largely unknown. Accumulating evidence implies that mitochondrial dysfunction may play an important role in schizophrenia^2^.

Human brain is the largest energy consumer among all organs (it consumes about 20% energy used by the human body)^3^, which makes it more susceptible to disrupted cellular energy metabolism. Most of the energy used by the human body is produced by mitochondrion, a complex intracellular organelle with many components. As the energy factory, mitochondrion plays a key role in maintaining the normal function of brain and mitochondrial dysfunction has been frequently reported in patients with brain diseases, including neurodegenerative diseases and psychiatric disorders^2^,^4^.

Several lines of evidence support the dysfunction of mitochondria in schizophrenia. First, defect of mitochondrial oxidative phosphorylation and altered expression of mitochondria-related genes were reported in brains from patients with schizophrenia^5^,^6^,^7^. Second, data from transcriptomics, proteomics and metabolomics revealed aberrant brain metabolism and oxidative stress in schizophrenia patients^8^. Third, perturbations in mitochondrial network dynamics and in complex I dependent cellular respiration were also reported in schizophrenia^9^. Besides, genetic variations of mitochondrial DNA (mtDNA) were also reported to be susceptible to schizophrenia and other neurological disorders^2^,^10^,^11^, although with controversies^12^,^13^,^14^,^15^. Despite the significant progress in recent years, the role of mitochondria in the pathophysiology of schizophrenia remains elusive, and further studies using more rigorous methodological and statistical standards are necessary to avoid false positive results.

Among the mitochondrial complexes, NADH ubiquinone oxidoreductase (complex I) may have a role in schizophrenia. Human mitochondrial complex I, which contains 45 subunits, is the largest and most complicated component of the respiratory chain and plays a central role in electron transportation^16^. The 14 “core” subunits of complex I are conserved from bacteria to human and are sufficient for catalysis^17^,^18^, suggesting their importance in energy metabolism. Seven of the 14 “core” subunits were nuclear-encoded which ligate the flavin mononucleotide and the iron-sulfur clusters^19^. Due to its pivotal role in energy metabolism, it has been speculated that mitochondrial complex I may be involved in the pathophysiology of schizophrenia. Consistent with this speculation, previous studies showed aberrant expression of mitochondrial complex I subunits or altered complex I activity in schizophrenia^6^,^20^,^21^,^22^. In addition, several “core” genes of mitochondrial complex I have been reported to be functionally related to schizophrenia^23^,^24^,^25^.

To further explore the potential association between mitochondrial complex I and schizophrenia, we conducted a comprehensive genetic association study by genotyping 46 tag SNPs from seven nuclear-encoded genes of mitochondrial complex I in Han Chinese with and without schizophrenia and reanalysis of the Psychiatric Genetics Consortium (PGC) dataset^26^. In addition, we also performed stratified analyses to test if genetic variants of the seven nuclear-encoded genes of mitochondrial complex I were associated with early onset schizophrenia (EOS) in Han Chinese.

## Results

In total, 46 tag SNPs were successfully genotyped in our samples. Considering an average population minor allele frequency (MAF) of 0.1, the power to detect an odds ratio as low as 1.5 for a risk allele/genotype/haplotype was above 90% for the comparison between schizophrenia patients and controls in our sample set (Supplementary Figure 1).

**Figure 1:**
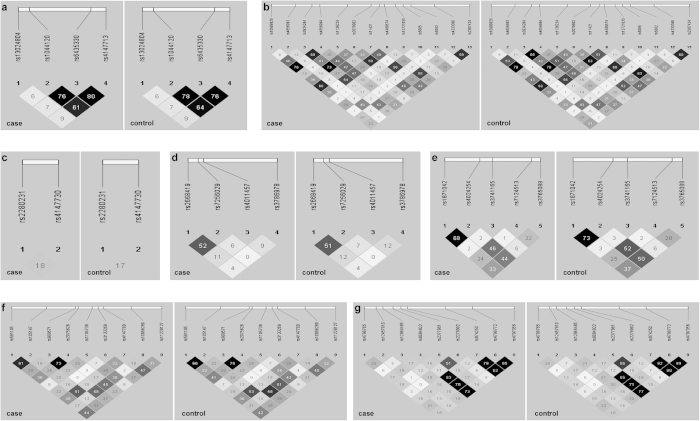
The linkage disequilibrium (LD) structures of the *NDUFS1* gene (a), the *NDUFS2* gene (b), the *NDUFS3* gene (c), the *NDUFS7* gene (d), the *NDUFV1* gene (e), the *NDUFS8* gene (f) and the *NDUFV2* gene (g) in Han Chinese with and without schizophrenia. The value in each square refers to *r*^*2*^ × 100. The blacker square represented the higher LD. The individual square showed the *r*^*2*^ × 100 value for each SNP pair.

None of the 46 SNPs was deviated from HWE in both case and control samples (Supplementary Table 1). The linkage disequilibrium (LD) structures of the 46 SNPs showed high degrees of similarity between cases and controls (Fig. 1). Allelic and genotypic association analyses revealed no significant association between these 46 tag SNPs and schizophrenia (Table 1). In addition, haplotype-based analysis also showed no significant association between schizophrenia cases and healthy controls (Supplementary Table 2). Further stratified association analysis revealed that two SNPs (rs4147713, *P* = 0.019; and rs1044120, *P *= 0.049) in the *NDUFS1* gene and 4 SNPs (rs3924264, *P* = 0.004; rs1136224, *P* = 0.013; rs2070902, *P* = 0.006; and rs4233368, *P* = 0.038) in the *NDUFS2* gene were nominally associated with EOS (onset age ≤ 18 years) (Table 2). Nevertheless, none of the six SNPs survived for multiple testing corrections.

We further examined the genetic association between these 46 SNPs and schizophrenia in the PGC data^26^. Overall, 35,476 schizophrenia cases and 46,839 controls were included for association analysis. We observed marginally significant associations with schizophrenia of rs10908826, rs4656994, rs5085 and rs2307424 in the *NDUFS2* gene and rs12457810, rs12964485 and rs2377961 in the *NDUFV2* gene. However, none of these SNPs showed significant association after correcting for multiple testing (Table 1).

We further performed interaction analysis to test whether there is an interaction between SNPs in both case-control samples and the EOS-control samples. Our results showed that although there were many marginally significant interactions, none of them could survive the Bonferroni correction (Supplementary Tables 3-4).

## Discussion

Mitochondrial dysfunction has been frequently reported in schizophrenia^8^,^27^. Previous studies have suggested that genes related to energy metabolism and oxidative stress may be responsible for mitochondrial dysfunction in schizophrenia^8^. As the largest component of the three membrane-bound enzymes, mitochondrial complex I plays a vital role in energy metabolism and altered activity of mitochondrial complex I was repeatedly reported in schizophrenia^20^,^22^,^28^,^29^. Though multiple studies have reported that mitochondrial dysfunction may be involved in schizophrenia pathogenesis, most of the conclusions were based on gene expression^6^,^21^. We previously analyzed the *NDUFS7* gene in Han Chinese with and without schizophrenia, with an intention to discern the effect of the complex I genes on this disorder^30^. In this study, we analyzed other complex I core subunit genes including *NDUFS1*, *NDUFS2*, *NDUFS3*, *NDUFS8*, *NDUFV1* and *NDUFV2*, as well as four more common SNPs of the *NDUFS7* gene in a larger sample set. We systematically investigated the potential association between these nuclear-encoded mitochondrial complex I genes and schizophrenia in a Chinese case-control sample. Our results revealed no significant association between genetic variants from the seven selected genes and schizophrenia, suggesting that these genes are unlikely to confer risk of schizophrenia in Han Chinese population. Consistent with the finding in Han Chinese, we also found no robust association between these SNPs and schizophrenia in the PGC data^26^. It should be mentioned that if we did not consider the effect of multiple corrections, SNPs of the *NDUFS2* gene would show (marginally) significant association with schizophrenia in both Han Chinese and the PGC data^26^.

It is interesting that we observed nominal significant association between genetic variants in the *NDUFS1* and *NDUFS2* genes and EOS in Han Chinese. The nominal significance would not survive from adjustment and we cannot rule out the possibility of false positive association caused by relatively small sample size of EOS used in the analyses. It should be mentioned that genetic variant in the *NDUFS1* gene was reported to be associated with schizophrenia and negative symptoms in Han Chinese from Eastern China, albeit the samples size was also relatively small^31^. We also noticed that comparing with whole samples (odds ratio [OR] > 1), 2 of the 6 nominal significant SNPs (rs4147713 and rs3924264) were associated with EOS in reverse style (OR < 1). This observation indicated population stratification may exist in the EOS-subpopulation.

Considering that mitochondrial dysfunction was repeatedly reported in schizophrenia, it is amazing that none of the selected SNPs showed significant association with schizophrenia. One of the possible explanations is that other genes but not the seven selected core genes of mitochondrial complex I contribute to schizophrenia susceptibility. Moreover, possibility of rare variant(s) in these genes contributing to schizophrenia susceptibility needs to be studied as well. In addition, given that schizophrenia has a strong genetic heterogeneity, it is also possible that these genes would have a strong effect in other populations but not in Han Chinese^23^,^24^.

There are several limitations in this study. First, the sample size is relatively modest in this study. As a result, it may be difficult to detect a robust significant association. Second, we only analyzed seven nuclear-encoded genes in the mitochondrial complex I in this study, we could not exclude the possibility that other genes of complex I are associated with schizophrenia.

In summary, we detected no significant association between the genetic polymorphisms of the nuclear-encoded core subunit genes of mitochondrial complex I and schizophrenia by using a rigorous statistical standard so as to avoid false positive results. Further work is needed to test if the expression and rare variants, but not common variants of these genes, contribute to schizophrenia.

## Materials and methods

### Subjects

A total of 1960 subjects, including 918 unrelated patients with schizophrenia (561 males: mean age ± SD, 38.5 ± 13.6 years; 357 females: 38.4 ± 16.8 years) and 1042 healthy controls (631 males: mean age ± SD, 38.5 ± 14.2 years; 411 females: 46.0 ± 8.2 years) were recruited. All of the individuals are of Han Chinese origin from Hunan Province of South Central China. Among the 918 patients, 189 were EOS (first age of onset ≤ 18 years old). The patients were clinically diagnosed according to Diagnostic and Statistical Manual of Mental Disorders IV (DSM-IV) and had at least two-year history of schizophrenia. Diagnosis and review of schizophrenia cases were independently checked and verified by two senior psychiatrists prior to blood sample collection. The healthy controls were collected from local hospitals and assessed by experienced psychiatrists. Individuals with psychiatric history, alcohol dependence, drug abuse, or family history of psychiatric disorders were excluded. All of the schizophrenia patients and healthy controls have been reported in our previous studies^11^,^32^. Written informed consents conforming to the tenets of the Declaration of Helsinki were obtained from all participants or the appointed guardians of the patients (for those who were unable to provide informed consent at the time of blood collection) prior to this study. The experimental methods were carried out in accordance with the approved guidelines. All experimental protocols of this study were approved by the institutional review board / Ethics Committee of Kunming Institute of Zoology, Chinese Academy of Sciences.

### SNP selection and genotyping

Genomic DNA of all participants was extracted from peripheral blood using the AxyPrep^TM^ Blood Genomic DNA Miniprep Kit (Axygen, USA) according to the manufacturer’s instruction. Seven nuclear-encoded genes of mitochondrial complex I (*NDUFS1*, *NDUFS2*, *NDUFS3*, *NDUFS7*, *NDUFS8*, *NDUFV1* and *NDUFV2*) were chosen in this study. To select the tag SNPs, we retrieved genotypic data of Han Chinese (CHB) from the HapMap database ( http://hapmap.ncbi.nlm.nih.gov/) and defined LD blocks using the Haploview 4.2^33^. The gene region and potential regulatory sequences (20 kb of both upstream and downstream regions) were taken into consideration during the selection of tag SNPs. In total, 51 tag SNPs were selected based on the following criteria: minor allele frequency (MAF) ≥ 0.1 and *r*^*2*^ ≥ 0.8 (Supplementary Figures 2-5). Three tag SNPs (rs2074896, rs2074897 and rs2074898) in the *NDUFS7* gene were analyzed in our previous study^30^, therefore were not included in this study. The remaining 48 tag SNPs were divided into four panels (12 SNPs for each panel) according to their compatibility in multiplex PCR. Genotyping of each panel was conducted by SNaPshot assays reported in our previous studies^34^,^35^. The GeneMarker software was utilized to read the genotyping results^36^.

### Power calculation and statistical analysis

Among the 48 tag SNPs, two (rs12798346 and rs3751084) were failed to be genotyped in our samples. Therefore, these two SNPs were excluded from our statistical analysis. The genotyping call rate of each SNP was above 99.0% in 1960 individuals. LD plot of the genotyped SNPs of each gene was constructed using Haploview 4.2 program (version 4.2). We tested deviation from the Hardy-Weinberg equilibrium (HWE), individual SNP association, haplotype comparison and SNP-SNP interaction by using PLINK^37^. Quanto software^38^ was used for power analysis under the gene only hypothesis and log additive model and following parameters: risk allele frequency from 0.1 to 0.5 in increments of 0.1; overall disease risk in the general population = 0.01; sample size = 918 cases vs. 1042 controls; range of OR from 1.0 to 2.0 in increments of 0.1; two-sided type I error rate = 0.05.

### PGC data analysis

To further explore if the studied SNPs are associated with schizophrenia, we extracted the genetic association data from the Psychiatric Genomics Consortium (PGC, http://www.broadinstitute.org/mpg/ricopili/)^39^ and reanalyzed this data set as an independent validation sample. In brief, 35,476 schizophrenia cases and 46,839 controls were included in the PGC dataset. The genotyping of each primary GWAS study composing the PGC data was performed by Affymetrix or Illumina array and the genetic association analysis was conducted by PLINK^37^ under an additive logistic regression model. More detailed information about the PGC can be found in the original publication^26^.

## Additional Information

**How to cite this article**: Li, X. *et al.* Do nuclear-encoded core subunits of mitochondrial complex I confer genetic susceptibility to schizophrenia in Han Chinese populations? *Sci. Rep.* **5**, 11076; doi: 10.1038/srep11076 (2015).

## Supplementary Material

Supplementary Information

## Figures and Tables

**Table 1:** **Association results of the 46 SNPs in Hunan sample and the PGC sample.**

**SNP ID**	**A**_**12**_^a^	**Freq.**^b^	***P-*****value**^c^		**OR (s.e.)**^d^	
			**Hunan**	**PGC**	**Hunan**	**PGC**
*NDUFS1*						
rs4147713	G/T	0.325	0.570	0.165	1.039 (0.068)	0.985 (0.011)
rs6435330	T/G	0.280	0.357	0.077	1.067 (0.071)	1.019 (0.011)
rs1044120	T/G	0.243	0.892	0.106	0.990 (0.075)	1.018 (0.011)
rs13024804	G/A	0.174	0.174	0.277	0.889 (0.086)	1.022 (0.020)
*NDUFS2*						
rs10908826	T/C	0.346	0.319	**0.028**	0.935 (0.068)	1.033 (0.015)
rs4656993	A/G	0.100	0.110	0.274	1.179 (0.103)	0.988 (0.011)
rs3924264	A/G	0.493	0.628	0.314	1.031 (0.064)	1.011 (0.011)
rs4656994	A/G	0.393	0.588	**0.016**	0.965 (0.066)	1.031 (0.013)
rs1136224	C/T	0.304	0.892	0.105	0.991 (0.070)	1.024 (0.015)
rs2070902	T/C	0.455	0.509	0.100	0.958 (0.064)	0.980 (0.012)
rs11421	C/T	0.384	0.312	0.059	0.935 (0.066)	0.973 (0.015)
rs4489574	T/C	0.474	0.826	0.057	1.014 (0.064)	0.979 (0.011)
rs12721035	A/G	0.146	0.579	0.406	1.051 (0.090)	1.018 (0.022)
rs5085	C/G	0.305	0.347	**0.015**	0.936 (0.070)	0.967 (0.014)
rs5082	C/T	0.088	0.149	0.585	1.171 (0.109)	1.006 (0.011)
rs4233368	A/C	0.398	0.306	0.066	0.935 (0.066)	0.978 (0.012)
rs2307424	C/T	0.491	0.885	**0.017**	0.991 (0.064)	1.027 (0.011)
*NDUFS3*						
rs2280231	T/C	0.278	0.846	0.167	0.986 (0.072)	0.984 (0.012)
rs4147730	A/G	0.318	0.721	0.311	1.025 (0.069)	1.015 (0.015)
*NDUFS7*						
rs2668419	A/G	0.438	0.873	0.383	1.010 (0.064)	1.017 (0.020)
rs7256029	G/A	0.406	0.881	0.354	0.990 (0.065)	1.017 (0.018)
rs4011457	C/G	0.145	0.987	0.143	1.002 (0.091)	0.960 (0.028)
rs3786978	C/T	0.395	0.836	0.196	1.014 (0.065)	1.044 (0.033)
*NDUFS8*						
rs581105	G/T	0.390	0.285	0.585	1.072 (0.065)	1.006 (0.011)
rs105147	C/T	0.465	0.462	0.865	1.049 (0.064)	1.002 (0.011)
rs999571	A/G	0.183	0.685	0.053	0.967 (0.083)	0.969 (0.017)
rs2075626	C/T	0.222	0.594	0.408	1.042 (0.077)	1.010 (0.013)
rs1104739	C/A	0.235	0.940	0.747	0.994 (0.076)	0.996 (0.011)
rs3133269	C/T	0.202	0.826	0.675	1.018 (0.080)	1.005 (0.012)
rs4147780	C/T	0.428	0.821	0.277	1.015 (0.065)	1.012 (0.011)
rs10896289	A/C	0.143	0.897	0.055	0.988 (0.092)	0.973 (0.015)
rs11228127	A/G	0.317	0.760	0.098	0.979 (0.069)	0.973 (0.016)
*NDUFV1*						
rs1871042	T/C	0.172	0.267	0.266	0.909 (0.086)	0.987 (0.012)
rs4024254	C/T	0.207	0.380	0.365	0.932 (0.080)	1.010 (0.011)
rs3741165	G/A	0.134	0.638	0.390	1.045 (0.093)	1.047 (0.053)
rs7124513	T/C	0.127	0.061	0.425	0.830 (0.100)	1.009 (0.012)
rs3765088	G/A	0.326	0.692	0.950	1.027 (0.068)	1.000 (0.011)
*NDUFV2*						
rs4798765	T/C	0.323	0.051	0.623	0.873 (0.069)	1.006 (0.011)
rs12457810	G/T	0.107	0.980	**0.035**	0.997 (0.104)	0.958 (0.021)
rs12964485	T/C	0.394	0.967	**0.019**	0.997 (0.066)	0.975 (0.011)
rs8084822	T/A	0.250	0.327	0.786	0.929 (0.075)	0.996 (0.014)
rs2377961	C/T	0.345	0.071	**0.019**	1.128 (0.067)	0.973 (0.012)
rs2279992	G/A	0.275	0.119	0.107	1.117 (0.071)	0.982 (0.011)
rs874250	A/G	0.261	0.250	0.538	0.919 (0.074)	1.008 (0.014)
rs4798772	G/A	0.299	0.342	0.507	0.935 (0.071)	1.008 (0.012)
rs4797356	A/T	0.283	0.497	0.163	0.953 (0.072)	1.015 (0.011)

^a^A_12_, minor allele and major allele.

^b^Minor allele frequency (Freq.) of control samples in Hunan.

^c^*P*-value < 0.05 was marked in bold.

^d^Odds ratio (OR) estimates and standard errors (s.e.).

**Table 2:** **Association results of the 46 SNPs in 189 early onset schizophrenia patients and 1042 controls.**

**SNP ID**	**A**_**12**_^a^	**Frequency of minor allele**		**OR**	**95% CI**	***P*****-value**^b^
		**EOS**	**control**			
*NDUFS1*						
rs4147713	G/T	0.265	0.325	0.746	0.583-0.954	**0.019**
rs6435330	T/G	0.246	0.280	0.838	0.651-1.079	0.171
rs1044120	T/G	0.196	0.243	0.760	0.579-0.999	**0.049**
rs13024804	G/A	0.204	0.174	1.213	0.922-1.596	0.168
*NDUFS2*						
rs10908826	T/C	0.373	0.346	1.122	0.894-1.409	0.319
rs4656993	A/G	0.130	0.100	1.336	0.958-1.863	0.087
rs3924264	A/G	0.426	0.507	0.721	0.578-0.899	**0.004**
rs4656994	A/G	0.434	0.393	1.184	0.948-1.477	0.136
rs1136224	C/T	0.241	0.304	0.727	0.564-0.937	**0.013**
rs2070902	T/C	0.378	0.455	0.729	0.582-0.913	**0.006**
rs11421	C/T	0.429	0.384	1.204	0.964-1.503	0.101
rs4489574	T/C	0.434	0.474	0.852	0.683-1.062	0.154
rs12721035	A/G	0.161	0.146	1.130	0.837-1.525	0.425
rs5085	C/G	0.336	0.305	1.152	0.913-1.454	0.234
rs5082	C/T	0.087	0.088	0.994	0.674-1.465	0.974
rs4233368	A/C	0.341	0.398	0.784	0.623-0.987	**0.038**
rs2307424	C/T	0.439	0.491	0.812	0.651-1.012	0.064
*NDUFS3*						
rs2280231	T/C	0.315	0.278	1.194	0.942-1.514	0.142
rs4147730	A/G	0.304	0.318	0.939	0.741-1.191	0.605
*NDUFS7*						
rs2668419	A/G	0.460	0.438	1.094	0.878-1.363	0.424
rs7256029	G/A	0.413	0.406	1.028	0.823-1.285	0.806
rs4011457	C/G	0.124	0.145	0.835	0.601-1.160	0.281
rs3786978	C/T	0.400	0.395	1.019	0.815-1.275	0.868
*NDUFS8*						
rs581105	G/T	0.378	0.390	0.951	0.759-1.192	0.665
rs105147	C/T	0.455	0.465	0.961	0.771-1.198	0.724
rs999571	A/G	0.156	0.183	0.824	0.611-1.111	0.204
rs2075626	C/T	0.201	0.222	0.881	0.671-1.156	0.361
rs1104739	C/A	0.217	0.235	0.903	0.693-1.176	0.447
rs3133269	C/T	0.217	0.202	1.097	0.840-1.433	0.497
rs4147780	C/T	0.426	0.428	0.992	0.794-1.237	0.940
rs10896289	A/C	0.122	0.143	0.834	0.598-1.162	0.282
rs11228127	A/G	0.307	0.317	0.953	0.752-1.208	0.692
*NDUFV1*						
rs1871042	T/C	0.169	0.172	0.979	0.732-1.311	0.889
rs4024254	C/T	0.201	0.207	0.965	0.735-1.268	0.799
rs3741165	G/A	0.143	0.134	1.078	0.787-1.477	0.639
rs7124513	T/C	0.101	0.127	0.767	0.536-1.099	0.147
rs3765088	G/A	0.347	0.326	1.095	0.869-1.379	0.441
*NDUFV2*						
rs4798765	T/C	0.275	0.323	0.794	0.622-1.013	0.063
rs12457810	G/T	0.101	0.107	0.933	0.649-1.341	0.707
rs12964485	T/C	0.384	0.394	0.955	0.763-1.197	0.691
rs8084822	T/A	0.228	0.250	0.886	0.683-1.149	0.361
rs2377961	C/T	0.386	0.345	1.197	0.955-1.501	0.118
rs2279992	G/A	0.309	0.275	1.178	0.928-1.497	0.179
rs874250	A/G	0.238	0.261	0.887	0.687-1.146	0.358
rs4798772	G/A	0.271	0.299	0.875	0.684-1.119	0.287
rs4797356	A/T	0.263	0.283	0.907	0.706-1.164	0.441

^a^A_12_, minor allele and major allele.

^b^*P*-value < 0.05 was marked in bold.

OR - odds ratio; 95% CI – 95% confidence interval
