# Supplementary material for: Do nuclear-encoded core subunits of mitochondrial complex I confer genetic susceptibility to schizophrenia in Han Chinese populations?
Source: Sci Rep. 2015 Jun 8;5:11076. doi: 10.1038/srep11076 (PMC4459149; doi:10.1038/srep11076)
Supplement: Supplementary Information [file srep11076-s1.doc]

**Online Supplementary File**

**Do nuclear-encoded core subunits of mitochondrial complex I confer genetic susceptibility to schizophrenia in Han Chinese populations?**

Xiao Li^1,3,a^, Wen Zhang^1,a^, Jinsong Tang^2^, Liwen Tan^2^, Xiong-jian Luo^1^, Xiaogang Chen^2,*^, Yong-Gang Yao^1,4*^

^1^Key Laboratory of Animal Models and Human Disease Mechanisms of the Chinese Academy of Sciences & Yunnan Province, Kunming Institute of Zoology, Chinese Academy of Sciences, Kunming 650223, China;

^2^Institute of Mental Health, the Second Xiangya Hospital, Central South University, Changsha 410011, China;

^3^Kunming College of Life Science, University of Chinese Academy of Sciences, Kunming 650223, China;

^4^CAS Center for Excellence in Brain Science, Chinese Academy of Sciences, Shanghai, 200031, China.

^a^These authors contributed equally to this work.

^*^Corresponding author: Dr. Yong-Gang Yao, Key Laboratory of Animal Models and Human Disease Mechanisms, Kunming Institute of Zoology, Chinese Academy of Sciences, Kunming, Yunnan 650223, China. Tel/Fax: 86-871-65180085; E-mail: yaoyg@mail.kiz.ac.cn; or Dr. Xiaogang Chen, Institute of Mental Health, the Second Xiangya Hospital, Central South University, Changsha 410011, China. E-mail: chenxghn@163.com

**Supplementary Table 1** Genotype frequencies of the 46 SNPs in 918 schizophrenia patients and 1042 healthy controls from Hunan Province.

| SNP ID | Genotype | Number of samples | | *P*-value^a^ | HWE *P*-value |
| --- | --- | --- | --- | --- | --- |
|  |  | case | control |  |  |
| *NDUFS1* |  |  |  |  |  |
| rs4147713 | GG/GT/TT | 90/433/395 | 115/448/479 | 0.170 | 0.075/0.525 |
| rs6435330 | TT/GT/GG | 69/401/448 | 78/428/536 | 0.482 | 0.112/0.591 |
| rs1044120 | TT/GT/GG | 55/329/528 | 64/376/599 | 0.990 | 0.717/0.613 |
| rs13024804 | GG/AG/AA | 22/246/650 | 35/293/714 | 0.328 | 0.902/0.452 |
| *NDUFS2* |  |  |  |  |  |
| rs10908826 | TT/CT/CC | 101/405/410 | 125/472/445 | 0.608 | 0.941/1.000 |
| rs4656993 | AA/AG/GG | 9/195/713 | 10/189/843 | 0.218 | 0.336/1.000 |
| rs3924264 | AA/AG/GG | 230/459/229 | 258/511/273 | 0.816 | 1.000/0.536 |
| rs4656994 | AA/AG/GG | 134/438/346 | 169/481/392 | 0.582 | 0.336/1.000 |
| rs1136224 | CC/CT/TT | 91/372/455 | 87/459/496 | 0.209 | 0.241/0.213 |
| rs2070902 | TT/CT/CC | 172/471/274 | 218/512/312 | 0.443 | 0.256/0.755 |
| rs11421 | CC/CT/TT | 124/428/366 | 154/492/396 | 0.597 | 1.000/0.948 |
| rs4489574 | TT/CT/CC | 217/442/259 | 249/489/304 | 0.851 | 0.290/ 0.062 |
| rs12721035 | AA/AG/GG | 21/235/656 | 29/245/767 | 0.442 | 1.000/0.082 |
| rs5085 | CC/CG/GG | 77/381/460 | 99/438/505 | 0.614 | 0.936/0.770 |
| rs5082 | CC/CT/TT | 7/172/739 | 8/167/867 | 0.285 | 0.470/1.000 |
| rs4233368 | AA/AC/CC | 128/445/345 | 172/485/385 | 0.282 | 0.442/0.365 |
| rs2307424 | CC/CT/TT | 208/481/229 | 256/511/275 | 0.327 | 0.165/0.536 |
| *NDUFS3* |  |  |  |  |  |
| rs2280231 | TT/CT/CC | 76/353/489 | 81/417/544 | 0.754 | 0.282/0.938 |
| rs4147730 | AA/AG/GG | 105/383/430 | 111/440/491 | 0.856 | 0.174/0.392 |
| *NDUFS7* |  |  |  |  |  |
| rs2668419 | AA/AG/GG | 183/443/292 | 210/493/339 | 0.913 | 0.547/0.209 |
| rs7256029 | GG/AG/AA | 166/409/343 | 185/476/381 | 0.882 | 0.024/0.095 |
| rs4011457 | CC/CG/GG | 26/215/676 | 17/269/756 | 0.110 | 0.084/0.261 |
| rs3786978 | CC/CT/TT | 149/433/336 | 148/527/367 | 0.254 | 0.630/0.069 |
| *NDUFS8* |  |  |  |  |  |
| rs581105 | GG/GT/TT | 159/429/330 | 165/483/394 | 0.570 | 0.339/0.398 |
| rs105147 | CC/CT/TT | 201/457/243 | 235/496/308 | 0.348 | 0.6401/0.191 |
| rs999571 | AA/AG/GG | 33/261/623 | 37/308/697 | 0.867 | 0.369/0.679 |
| rs2075626 | CC/CT/TT | 55/311/552 | 51/361/630 | 0.555 | 0.224/1.000 |
| rs1104739 | CC/AC/AA | 57/313/543 | 66/357/618 | 0.996 | 0.196/0.143 |
| rs3133269 | CC/CT/TT | 39/297/581 | 46/327/666 | 0.903 | 0.919/0.500 |
| rs4147780 | CC/CT/TT | 179/431/304 | 192/508/342 | 0.729 | 0.252/0.899 |
| rs10896289 | AA/AC/CC | 23/213/682 | 26/245/771 | 0.987 | 0.219/0.207 |
| rs11228127 | AA/AG/GG | 92/390/436 | 102/457/483 | 0.829 | 0.759/0.721 |
| *NDUFV1* |  |  |  |  |  |
| rs1871042 | TT/CT/CC | 23/246/649 | 33/293/716 | 0.513 | 1.000/0.664 |
| rs4024254 | CC/CT/TT | 34/291/593 | 43/345/654 | 0.677 | 0.916/0.850 |
| rs3741165 | GG/AG/AA | 15/225/677 | 24/231/787 | 0.293 | 0.580/0.180 |
| rs7124513 | TT/CT/CC | 8/182/728 | 17/231/794 | 0.130 | 0.491/1.000 |
| rs3765088 | GG/AG/AA | 104/402/412 | 117/446/479 | 0.886 | 0.710/0.398 |
| *NDUFV2* |  |  |  |  |  |
| rs4798765 | TT/CT/CC | 72/395/448 | 109/456/477 | 0.099 | 0.266/1.000 |
| rs12457810 | GG/GT/TT | 12/172/734 | 11/201/830 | 0.841 | 0.602/0.872 |
| rs12964485 | TT/CT/CC | 150/423/345 | 158/506/378 | 0.525 | 0.300/0.650 |
| rs8084822 | TT/AT/AA | 56/319/538 | 60/399/581 | 0.293 | 0.359/0.457 |
| rs2377961 | CC/CT/TT | 122/438/356 | 126/466/450 | 0.149 | 0.525/0.784 |
| rs2279992 | GG/AG/AA | 81/382/452 | 86/391/548 | 0.197 | 1.000/ 0.182 |
| rs874250 | AA/AG/GG | 57/335/526 | 66/411/565 | 0.376 | 0.721/0.470 |
| rs4798772 | GG/AG/AA | 79/364/474 | 92/438/512 | 0.519 | 0.465/0.941 |
| rs4797356 | AA/AT/TT | 77/342/489 | 79/431/531 | 0.230 | 0.132/0.542 |

^a^*P* value were calculated by the Chi-square test.

**Supplementary Table 2** Haplotype frequencies of the 46 SNPs in the 7 genes in 918 schizophrenia patients and 1042 healthy controls from Hunan Province.

| Gene | Haplotype | Freq. | | *P*-value |
| --- | --- | --- | --- | --- |
|  |  | case | control |  |
| *NDUFS1* | TGGG | 0.157 | 0.174 | 0.171 |
|  | GTTA | 0.241 | 0.239 | 0.895 |
|  | GTGA | 0.051 | 0.039 | 0.070 |
|  | GGGA | 0.043 | 0.047 | 0.553 |
|  | TGGA | 0.508 | 0.502 | 0.684 |
| *NDUFS2* | TGAATCCCAGTCT | 0.093 | 0.086 | 0.496 |
|  | CGGGTTTTGGTAC | 0.106 | 0.116 | 0.393 |
|  | TGAATCCCGCTCT | 0.186 | 0.210 | 0.093 |
|  | CGGGCTTTGGTCT | 0.055 | 0.043 | 0.113 |
|  | CAAGTCTCGGCCC | 0.082 | 0.074 | 0.353 |
|  | TGAATCTTGGTAC | 0.060 | 0.053 | 0.355 |
|  | CGGGTTCCGCTCT | 0.055 | 0.049 | 0.394 |
|  | CGGGTCCCGCTCT | 0.031 | 0.030 | 0.804 |
|  | CGAATCTCAGTCT | 0.025 | 0.026 | 0.864 |
|  | CGGGCTTTGGTAC | 0.208 | 0.226 | 0.197 |
|  | CAAGTCTTGGTCT | 0.033 | 0.022 | 0.048 |
|  | CGAATCTTGGTAC | 0.017 | 0.017 | 0.951 |
|  | CGGGCTTCAGTCT | 0.015 | 0.014 | 0.909 |
|  | CGGGCTCCGCTCT | 0.018 | 0.022 | 0.375 |
|  | TGAATCTCGGCCC | 0.014 | 0.012 | 0.661 |
| *NDUFS3* | CA | 0.323 | 0.318 | 0.721 |
|  | TG | 0.275 | 0.278 | 0.846 |
|  | CG | 0.402 | 0.404 | 0.871 |
| *NDUFS7* | GACC | 0.114 | 0.120 | 0.573 |
|  | GGGC | 0.148 | 0.145 | 0.796 |
|  | AAGC | 0.127 | 0.122 | 0.623 |
|  | GAGC | 0.018 | 0.014 | 0.353 |
|  | GACT | 0.021 | 0.018 | 0.584 |
|  | GGGT | 0.252 | 0.259 | 0.633 |
|  | AAGT | 0.320 | 0.322 | 0.906 |
| *NDUFS8* | GCGTATTCG | 0.036 | 0.027 | 0.157 |
|  | GCACATTCG | 0.023 | 0.023 | 0.976 |
|  | GCACCTCAA | 0.115 | 0.126 | 0.329 |
|  | TTGTATTCG | 0.516 | 0.516 | 0.973 |
|  | GCGCATTCG | 0.015 | 0.016 | 0.889 |
|  | TTGTATTCA | 0.009 | 0.013 | 0.350 |
|  | TCGTATTCG | 0.014 | 0.011 | 0.359 |
|  | TTGTACCCA | 0.015 | 0.023 | 0.082 |
|  | GCGTACCCA | 0.120 | 0.106 | 0.222 |
|  | GCGTACCCG | 0.037 | 0.042 | 0.494 |
|  | GCACACCCA | 0.025 | 0.020 | 0.350 |
|  | TCGTCTCCG | 0.063 | 0.065 | 0.810 |
|  | TCGTCTCCA | 0.012 | 0.011 | 0.897 |
| *NDUFV1* | TCATG | 0.072 | 0.086 | 0.102 |
|  | CCATG | 0.034 | 0.039 | 0.441 |
|  | TCACG | 0.081 | 0.079 | 0.791 |
|  | CTACG | 0.141 | 0.120 | 0.054 |
|  | CTGCA | 0.136 | 0.133 | 0.765 |
|  | CTACA | 0.535 | 0.542 | 0.641 |
| *NDUFV2* | CTTATAGAT | 0.259 | 0.258 | 0.930 |
|  | TTTATAGAT | 0.092 | 0.106 | 0.161 |
|  | CTCACAGAT | 0.032 | 0.030 | 0.649 |
|  | CTCACGGAT | 0.261 | 0.241 | 0.169 |
|  | CTCATAGAT | 0.040 | 0.040 | 0.984 |
|  | CTTACAGAT | 0.044 | 0.037 | 0.290 |
|  | TGCTTAAGA | 0.074 | 0.071 | 0.692 |
|  | CTCTTAAGA | 0.093 | 0.099 | 0.501 |
|  | TTCTTAAGA | 0.065 | 0.078 | 0.146 |
|  | TGCACGGAT | 0.019 | 0.022 | 0.532 |
|  | TTCATGGGA | 0.020 | 0.017 | 0.641 |

*NDUFS1*: rs4147713|rs6435330|rs1044120|rs13024804.

*NDUFS2*: rs10908826|rs4656993|rs3924264|rs4656994|rs1136224|rs2070902|rs11421|rs4489574|rs12721035|rs5085|rs5082|rs4233368|rs2307424.

*NDUFS3*: rs2280231|rs4147730.

*NDUFS7*: rs2668419|rs7256029|rs4011457|rs3786978.

*NDUFS8*: rs581105|rs105147|rs999571|rs2075626|rs1104739|rs3133269|rs4147780|rs10896289|rs11228127.

*NDUFV1*: rs1871042|rs4024254|rs3741165|rs7124513|rs3765088.

*NDUFV2*: rs4798765|rs12457810|rs12964485|rs8084822|rs2377961|rs2279992|rs874250|rs4798772|rs4797356.

**Supplementary Table 3** SNP-SNP interaction in 918 schizophrenia patients and 1042 controls.

| CHR1 | SNP1 | CHR2 | SNP2 | OR_INT^a^ | STAT^b^ | *P*-value^c^ |
| --- | --- | --- | --- | --- | --- | --- |
| 1 | rs10908826 | 1 | rs2307424 | 1.292 | 6.402 | 0.011 |
| 1 | rs3924264 | 1 | rs2307424 | 1.255 | 6.189 | 0.013 |
| 1 | rs3924264 | 2 | rs1044120 | 0.758 | 6.621 | 0.010 |
| 1 | rs3924264 | 2 | rs6435330 | 0.7912 | 5 | 0.025 |
| 1 | rs3924264 | 2 | rs4147713 | 0.7822 | 6.308 | 0.012 |
| 1 | rs3924264 | 19 | rs7256029 | 0.8338 | 4.1 | 0.043 |
| 1 | rs4656994 | 1 | rs2307424 | 1.273 | 6.324 | 0.012 |
| 1 | rs4656994 | 2 | rs1044120 | 0.795 | 4.454 | 0.035 |
| 1 | rs1136224 | 1 | rs2307424 | 0.8022 | 4.589 | 0.032 |
| 1 | rs1136224 | 2 | rs1044120 | 1.265 | 3.89 | 0.049 |
| 1 | rs1136224 | 2 | rs6435330 | 1.317 | 5.708 | 0.017 |
| 1 | rs1136224 | 2 | rs4147713 | 1.298 | 5.863 | 0.015 |
| 1 | rs1136224 | 18 | rs2279992 | 1.253 | 4.395 | 0.036 |
| 1 | rs2070902 | 1 | rs2307424 | 0.8247 | 4.337 | 0.037 |
| 1 | rs2070902 | 2 | rs1044120 | 1.287 | 5.194 | 0.023 |
| 1 | rs2070902 | 2 | rs4147713 | 1.244 | 4.779 | 0.029 |
| 1 | rs4489574 | 1 | rs2307424 | 0.7807 | 7.53 | 0.006 |
| 1 | rs4489574 | 2 | rs4147713 | 1.226 | 4.58 | 0.032 |
| 1 | rs4489574 | 19 | rs4011457 | 0.7614 | 4.809 | 0.028 |
| 1 | rs12721035 | 1 | rs2307424 | 1.357 | 3.869 | 0.049 |
| 1 | rs5082 | 19 | rs4011457 | 1.559 | 3.842 | 0.050 |
| 1 | rs4233368 | 11 | rs3133269 | 1.273 | 4.336 | 0.037 |
| 1 | rs4233368 | 19 | rs7256029 | 1.256 | 6.289 | 0.012 |
| 1 | rs4233368 | 19 | rs4011457 | 0.7309 | 5.693 | 0.017 |
| 1 | rs2307424 | 18 | rs12457810 | 1.392 | 4.774 | 0.029 |
| 1 | re2307424 | 19 | rs7256029 | 1.199 | 4.028 | 0.045 |
| 2 | rs13024804 | 11 | rs2280231 | 1.549 | 10.6 | 0.001 |
| 11 | rs4147730 | 11 | rs3765088 | 1.494 | 14.95 | 1.102x10^-4^ |
| 11 | rs4147730 | 11 | rs4147780 | 1.323 | 8.097 | 0.004 |
| 11 | rs4147730 | 11 | rs10896289 | 1.321 | 4.056 | 0.044 |
| 11 | rs3741165 | 19 | rs4011457 | 0.5953 | 6.781 | 0.009 |
| 11 | rs581105 | 18 | rs4798765 | 1.27 | 5.602 | 0.018 |
| 11 | rs581105 | 18 | rs2377961 | 0.8048 | 5.037 | 0.025 |
| 11 | rs581105 | 18 | rs4798772 | 1.238 | 4.523 | 0.033 |
| 11 | rs581105 | 18 | rs4797356 | 1.238 | 4.369 | 0.037 |
| 11 | rs105147 | 18 | rs2377961 | 0.7881 | 6.069 | 0.014 |
| 11 | rs105147 | 18 | rs874250 | 1.242 | 4.168 | 0.041 |
| 11 | rs105147 | 18 | rs4798772 | 1.252 | 4.925 | 0.026 |
| 11 | rs999571 | 19 | rs4011457 | 1.473 | 5.565 | 0.018 |
| 11 | rs2075626 | 18 | rs2377961 | 0.8063 | 3.867 | 0.049 |
| 18 | rs4798765 | 19 | rs3786978 | 0.81 | 4.039 | 0.044 |
| 18 | rs12964485 | 19 | rs2668419 | 1.248 | 5.697 | 0.017 |
| 18 | rs12964485 | 19 | rs3786978 | 0.8152 | 4.577 | 0.032 |
| 18 | rs2279992 | 19 | rs3786978 | 1.242 | 4.279 | 0.039 |

^a^OR_INT, Odds ratio for interaction.

^b^STAT, Chi-square statistic, 1df.

^c^The significant level for *P*-value should be 4.8x10^-5^ based on Bonferroni correction for multiple test (1035 tests in this analysis, only *P*-values less than 0.05 were shown).

**Supplementary Table 4** SNP-SNP interaction in 189 EOS patients and 1042 controls.

| CHR1 | SNP1 | CHR2 | SNP2 | OR_INT^a^ | STAT^b^ | *P*-value^c^ |
| --- | --- | --- | --- | --- | --- | --- |
| 1 | rs1136224 | 1 | rs5085 | 0.5819 | 4.71 | 0.030 |
| 1 | rs1136224 | 1 | rs6435330 | 1.588 | 4.547 | 0.033 |
| 1 | rs2070902 | 18 | rs4797356 | 1.446 | 4.309 | 0.038 |
| 1 | rs4489574 | 1 | rs5085 | 0.6596 | 4.202 | 0.040 |
| 1 | rs4489574 | 19 | rs4011457 | 0.6216 | 4.143 | 0.042 |
| 1 | rs5082 | 2 | rs13024804 | 2.297 | 6.356 | 0.012 |
| 1 | rs5082 | 11 | rs1104739 | 1.927 | 4.684 | 0.030 |
| 1 | rs2307424 | 2 | rs13024804 | 1.608 | 6.062 | 0.014 |
| 11 | rs2280231 | 11 | rs999571 | 1.593 | 4.367 | 0.037 |
| 11 | rs2280231 | 11 | rs11228127 | 1.497 | 4.732 | 0.030 |
| 11 | rs2280231 | 19 | rs3786978 | 1.526 | 5.874 | 0.015 |
| 11 | rs4147730 | 11 | rs3765088 | 1.71 | 8.882 | 0.003 |
| 11 | rs1871042 | 18 | rs2279992 | 0.5358 | 6.864 | 0.009 |
| 11 | rs4024254 | 11 | rs3133269 | 1.671 | 4.911 | 0.027 |
| 11 | rs4024254 | 11 | rs4147780 | 1.535 | 4.269 | 0.039 |
| 11 | rs4024254 | 11 | rs11228127 | 1.588 | 4.714 | 0.030 |
| 11 | rs4024254 | 18 | rs12964485 | 1.488 | 3.842 | 0.050 |
| 11 | rs4024254 | 18 | rs2279992 | 0.4341 | 12.38 | 4.35 x10^-4^ |
| 11 | rs3741165 | 19 | rs7256029 | 1.627 | 4.848 | 0.028 |
| 11 | rs3765088 | 18 | rs12964485 | 1.423 | 4.236 | 0.040 |
| 11 | rs3765088 | 18 | rs2279992 | 0.6356 | 6.483 | 0.011 |
| 11 | rs581105 | 11 | rs1104739 | 0.6657 | 4.237 | 0.040 |
| 11 | rs999571 | 11 | rs10896289 | 0.3471 | 6.643 | 0.010 |
| 11 | rs999571 | 19 | rs4011457 | 2.236 | 7.885 | 0.005 |
| 11 | rs2075626 | 11 | rs10896289 | 0.4381 | 4.254 | 0.039 |
| 18 | rs4798765 | 19 | rs4011457 | 0.4837 | 6.018 | 0.014 |
| 18 | rs12964485 | 19 | rs2668419 | 1.589 | 7.915 | 0.005 |
| 18 | rs12964485 | 19 | rs7256029 | 0.5992 | 9.866 | 0.002 |
| 18 | rs874250 | 19 | rs7256029 | 1.475 | 4.438 | 0.035 |
| 18 | rs4798772 | 19 | rs7256029 | 1.497 | 5.389 | 0.020 |
| 18 | rs4797356 | 19 | rs7256029 | 1.549 | 5.924 | 0.015 |
| 18 | rs4797356 | 19 | rs4011457 | 0.5294 | 4.047 | 0.044 |

^a^OR_INT, Odds ratio for interaction.

^b^STAT, Chi-square statistic, 1df.

^c^The significant level for *P*-value should be 4.8x10^-5^ based on Bonferroni correction for multiple test (1035 tests in this analysis, only *P*-values less than 0.05 were shown).


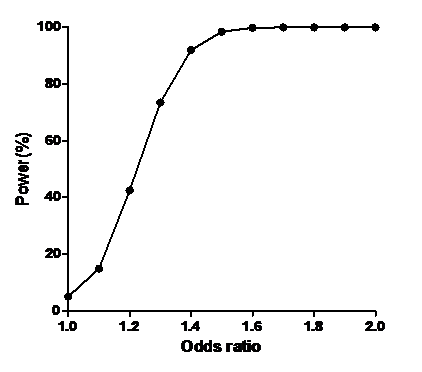


**Supplementary Figure 1** Power estimates for the case-control association analysis. Statistical power was computed under the gene only hypothesis and log additive model, with the following parameters: risk allele frequency = 0.1; overall disease risk in the general population = 0.01; sample size = 918 cases vs. 1042 controls; range of OR from 1.0 to 2.0 in increments of 0.1; two-sided type I error rate = 0.05.


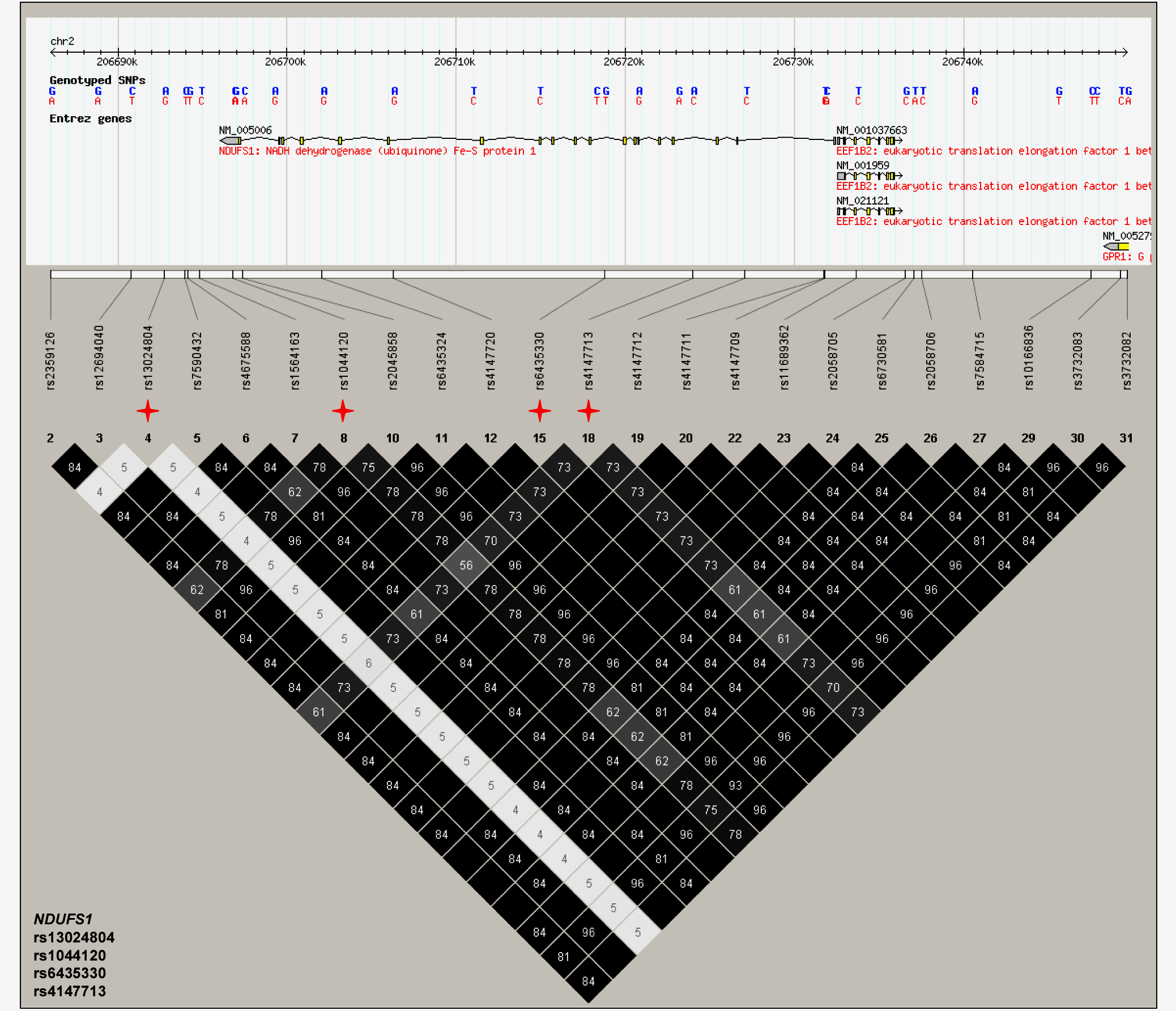


**Supplementary Figure 2** Linkage disequilibrium (LD) pattern of the *NDUFS1* gene (Genomic span: Chr2: 206,696,049 to 206,732,432 according to HapMap Data Rel 28 (same as below)) in CHB population from the HapMap database. The genotyped SNPs in this study were marked by red asterisks.


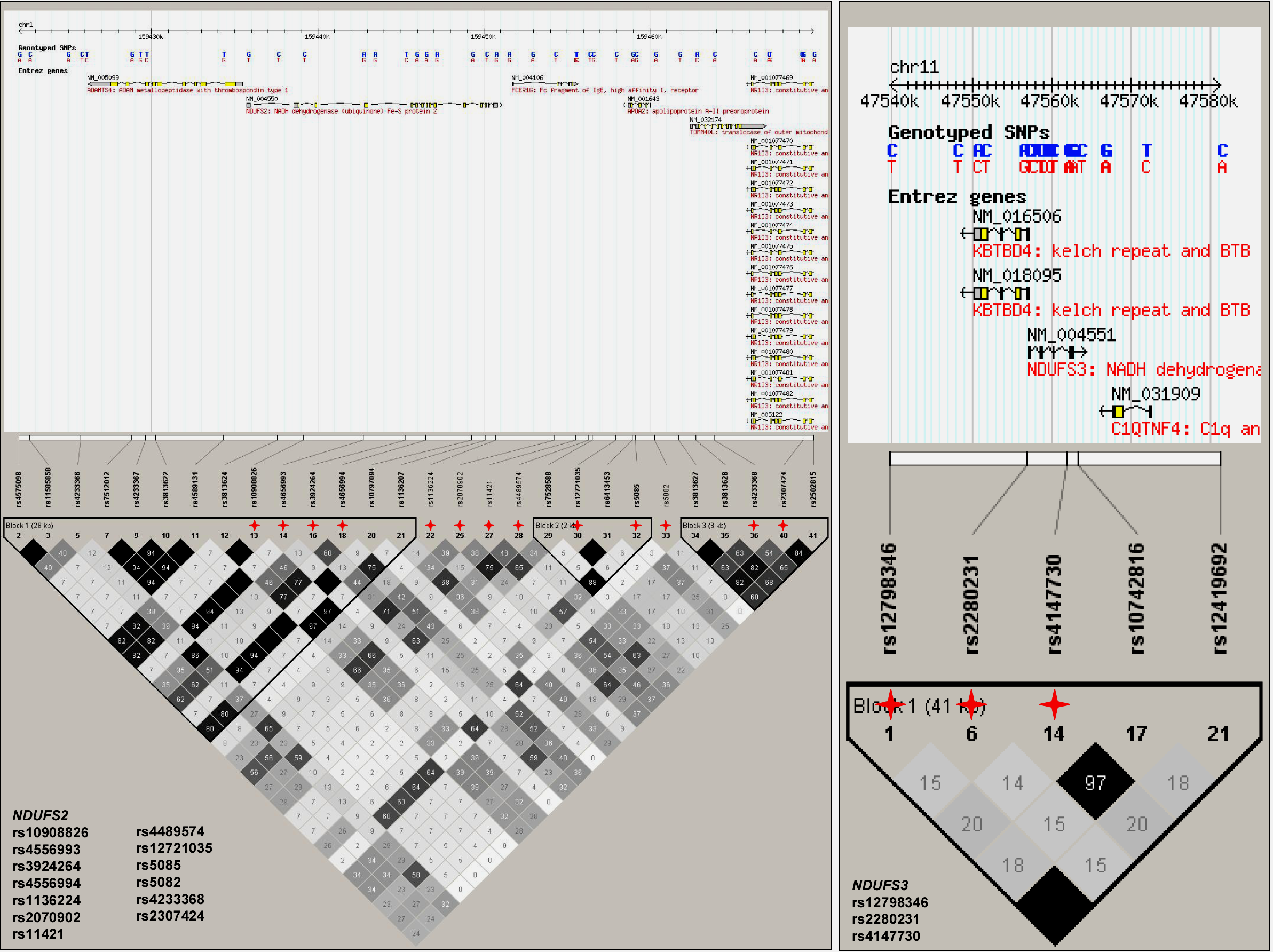


**Supplementary Figure 3** Linkage disequilibrium (LD) pattern of the *NDUFS2* (Genomic span: Chr1: 159,435,729 to 159,450,806) and *NDUFS3* (Genomic span: Chr11: 47,557,208 to 47,562,689) genes in CHB population from the HapMap database. The genotyped SNPs in this study were marked by red asterisks.


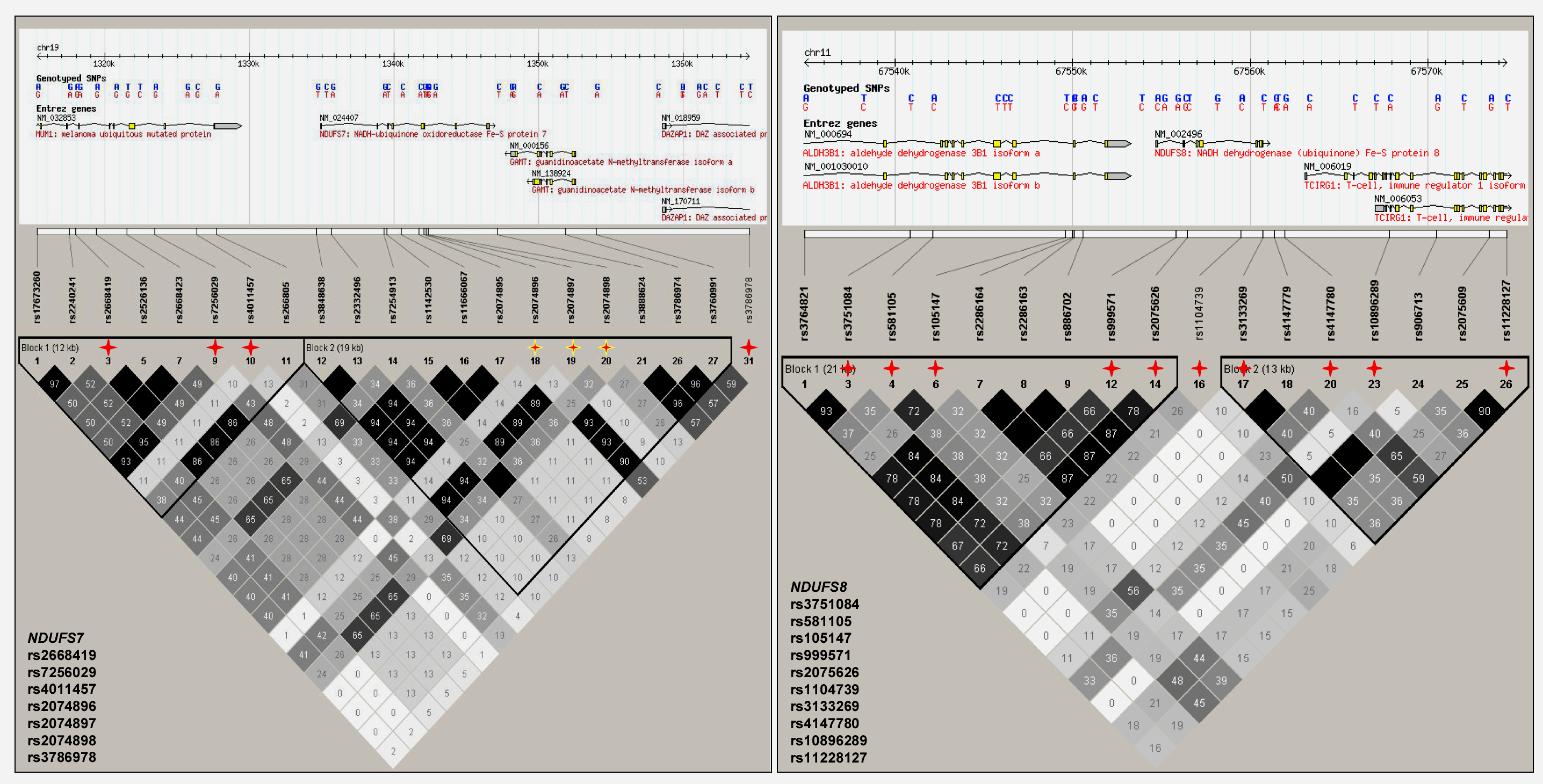


**Supplementary Figure 4** Linkage disequilibrium (LD) pattern of the *NDUFS7* (Genomic span: Chr19: 1,334,906 to 1,346,582) and *NDUFS8* (Genomic span: Chr11: 67,554,685 to 67,560,690) genes in CHB population from the HapMap database. The genotyped SNPs in this study were marked by red asterisks. Three SNPs (rs2074896, rs2074897 and rs2074898) of the *NDUFS7* gene marked in yellow asterisks were genotyped in our previous study and were not included in the current study.

**
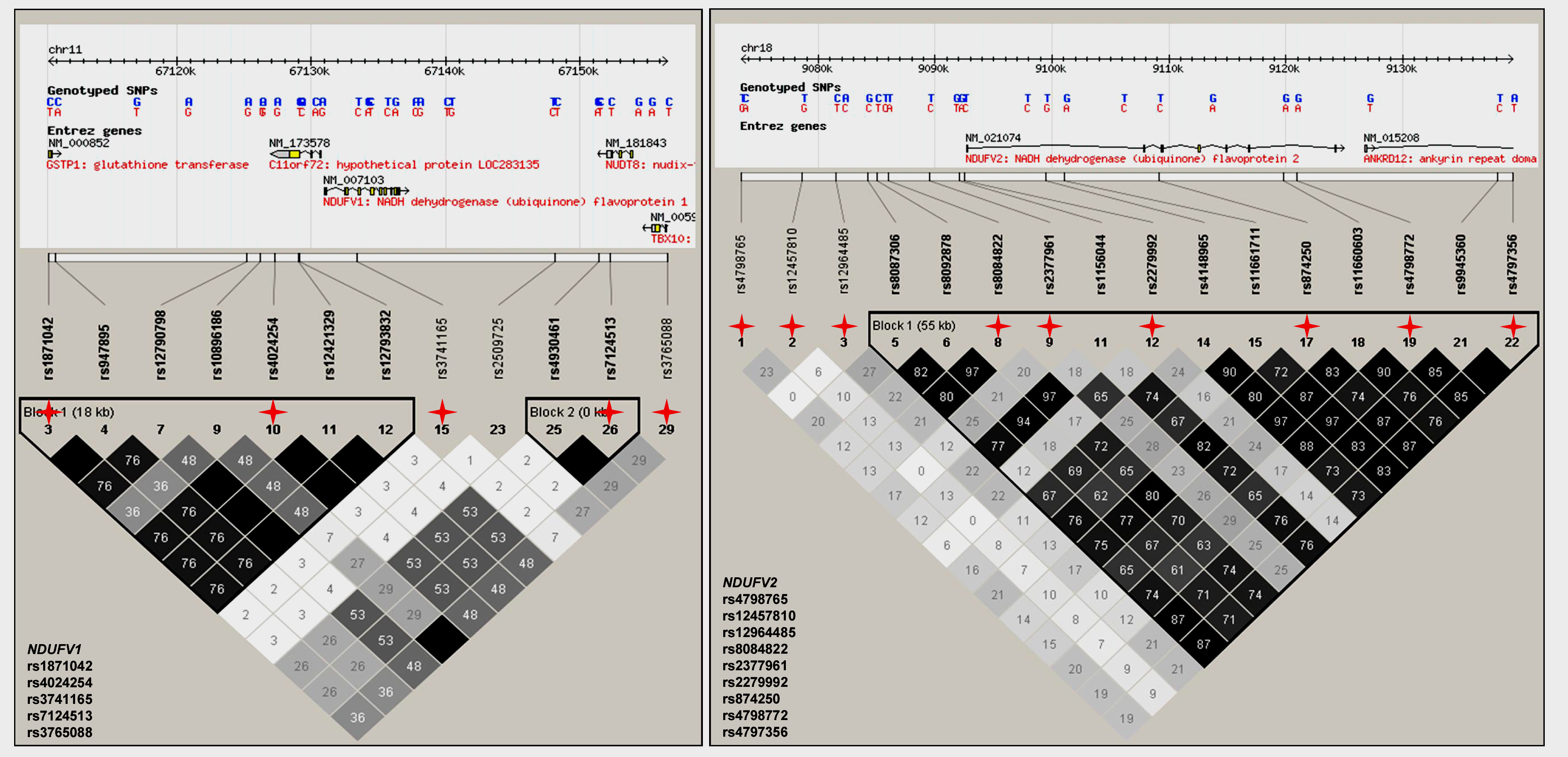
**

**Supplementary Figure 5** Linkage disequilibrium (LD) pattern of the *NDUFV1* (Genomic span: Chr11: 67,130,983 to 67,136,581) and *NDUFV2* (Genomic span: Chr18: 9,092,725 to 9,124,336) genes in CHB population from the HapMap database. The genotyped SNPs in this study were marked by red asterisks.
